# Intake of dairy protein during pregnancy in IBD and risk of SGA in a Norwegian population-based mother and child cohort

**DOI:** 10.1186/s12876-020-1182-y

**Published:** 2020-02-03

**Authors:** May-Bente Bengtson, Margaretha Haugen, Anne Lise Brantsæter, Geir Aamodt, Morten H. Vatn

**Affiliations:** 1 EpiGen-Institute, Faculty Division Akershus University Hospital, University of Oslo, Oslo, Norway; 2 Medical Department, Vestfold Hospital Trust, P.O. Box 2168, 3103 Tønsberg, Norway; 3 Division of Infection Control and Environmental Health, Department of Environmental Exposure and Epidemiology, Norwegian Institute of Public Health, P.O. Box 222-Skøyen, 0213 Oslo, Norway; 4 Department of Public Health Science, LANDSAM, Norwegian University of Life Sciences, P.O. Box 5003, 1432 Ås, Norway

**Keywords:** The Norwegian mother, Father and child cohort study (MoBa), Protein intake during pregnancy, SGA, Inflammatory bowel diseases, Inadequate GWG

## Abstract

**Background:**

Patients with inflammatory bowel disease (IBD) tend to avoid dairy products to minimize abdominal pain and diarrhea. The aim of this study was to estimate the proportion of protein from dairy sources (PPDS) in mothers with and without IBD, and to explore the impact of PPDS on inadequate gestational weight gain (GWG) or small for gestational age (SGA) in IBD compared to non-IBD in the population-based Norwegian Mother, Father and Child Cohort Study (MoBa).

**Methods:**

MoBa includes about 95,000 pregnant women recruited throughout Norway from 1999 to 2008. IBD phenotype and complications during pregnancy and delivery were ascertained. This study included 148 mothers with Crohn disease (CD) and 194 with ulcerative colitis and 68,858 non-IBD mothers. In mid-pregnancy participants answered a comprehensive semi-quantitative food frequency questionnaire assessing diet since the start of pregnancy. PPDS was ranked in quartiles. The two lowest quartiles were merged and considered to represent the lowest of three PPDS groups. We used logistic regression analyses to model multivariate associations, adjusting for potential confounders.

**Results:**

The risk of belonging to the lowest PPDS group was twice as high in IBD mothers compared to non-IBD mothers (aOR = 2.02, 95% CI: 1.53, 2.67). Low compared to high PPDS strongly predicted inadequate GWG in CD (aOR = 4.22, 95% CI: 1.28, 13.92). Surprisingly, and in opposition to the non-IBD mothers, PPDS was positively associated with the risk of SGA in IBD mothers. IBD mother with low PPDS was associated with significantly lower risk of SGA than non-IBD mothers and IBD mothers with high PPDS (aOR = 0.19, 95% CI: 0.07, 0.50). The interaction term IBD/PPDS was the factor that linked SGA to IBD compared to non-IBD, and increased the association between IBD and SGA with a factor of three.

**Conclusion:**

This study shows that intake of dairy products is lower in IBD mothers than in non-IBD mothers, and further, that low intake of dairy products in IBD mothers is associated with reduced risk of SGA compared to non-IBD and IBD mothers with high PPDS.

## Background

Inflammatory bowel disease (IBD) represents chronic complex disorders of the gastrointestinal tract, ulcerative colitis (UC) and Crohn’s disease (CD), with the highest incidence peak in fertile age. Mothers with IBD have an overall risk of adverse pregnancy outcomes with disease activity as the strongest predictor [1, 2], however, having IBD has been demonstrated to be a risk factor of its own [3–5]. The most consistent adverse pregnancy outcomes described are small for gestational age (SGA) [6], preterm birth (< 37 weeks of gestation) and low birth weight (< 2500 g) [7–9]. Weight loss and malnutrition are common features in IBD patients [10–12] and are well-known risk factors of SGA and low birth weight in the general population [13, 14]. Diarrhea, intestinal inflammation and bowel resections are all contributing factors to increased loss and impaired absorption of nutrition from the intestine in IBD. Maternal gestational weight gain (GWG) has a significant effect on fetal development and growth in the general population and in IBD [13–15]. Protein, and in particular consumption of protein from milk or dairy products, has a positive impact on mothers’ GWG as well as the infants’ birth weight [16].

Knowing that food restriction and avoidance of certain food products, especially dairy products [10, 17], is the main cause of weight loss and malnutrition in IBD patients, we hypothesized that IBD mothers had lower intake of dairy products than non-IBD, and furthermore, that reduced intake of dairy products increased the risk of both inadequate GWG and SGA in IBD mothers. The purpose of the present study was to examine: (i) the proportion of protein from dairy sources (PPDS) in IBD and non-IBD mothers, (ii) the impact of PPDS on inadequate GWG in IBD compared to non-IBD mothers (iii) the impact of PPDS on the association between SGA and IBD compared to non-IBD in a large population-based pregnancy cohort in Norway.

## Methods

### The Norwegian mother, father and child cohort study

The Norwegian Mother, Father and Child Cohort Study (MoBa) is a prospective population-based pregnancy cohort study conducted by the Norwegian Institute of Public Health [18]. The cohort includes 114,500 children and 95,200 mothers recruited from all over Norway in the period 1999 to 2008. The women were invited to the study by postal invitation prior to the first routine ultrasound examination in gestational weeks 17–20. Informed consent was obtained from all participants and they were asked to fill out comprehensive questionnaires at regular intervals. Four questionnaires were included in the present study, three during the pregnancy (Q1 – Q3) and one six months postpartum (Q4). The baseline questionnaire (Q1), answered around week 15–17, shortly before the first ultrasound visit, included information on mothers’ socio-demographic data, such as education, age, height, prepregnancy weight, health, lifestyle, and pregnancy complications. The second questionnaire (Q2) in week 22 was a food frequency questionnaire (FFQ) to obtain information on dietary habits and dietary supplement use in the first half of the pregnancy. The questionnaire Q3 in gestational week 30, included general background information and details on previous and present health problems and exposures. The questionnaire Q4 6 months after delivery included information about maternal and child anthropometrics, health and lifestyle at delivery and in the postpartum period. The cohort database is linked to pregnancy and birth records from the Medical Birth Registry of Norway (MBRN) [18].

## Materials

Participants had to have responded to Q1, Q2 and Q4 and be registered in MBRN with a singleton delivery to be eligible for inclusion in the current study. In total, *n* = 84,412 women fulfilled these criteria. In Q4, the participants reported weight at delivery and at 6 months postpartum. We only included women with singleton deliveries and GWG more than − 30 kg and less than 50 kg [14]. Of 739 mothers who claimed to suffer from IBD in the baseline questionnaire (Q1), only 655 had responded to the other questionnaires and were available for the present study. In 2013, these IBD mothers (655) received an invitation letter and a questionnaire to obtain detailed information about the IBD history.

**Fig. 1:**
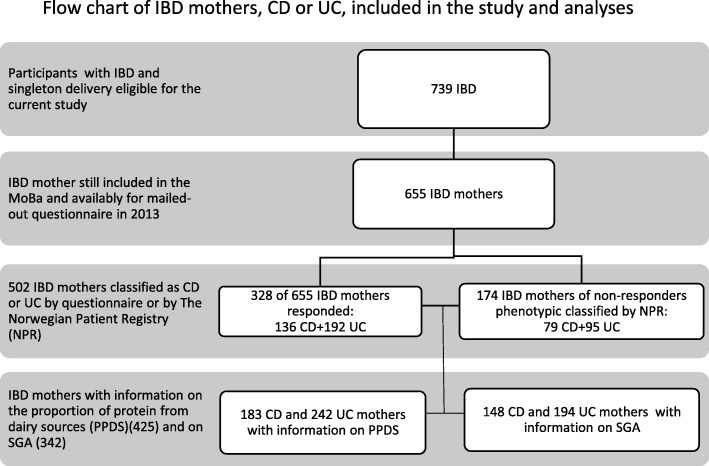
Flow chart of IBD mothers, CD or UC, included in the study and analyses. Legend: Flow chart of IBD mothers eligible for the study and the analyses. Two sources defined the phenotype CD and UC; respondents of the mail-out questionnaire in 2013 and The Norwegian Patient Registry (NPR)

Five hundred and two mothers were included with the diagnosis of IBD. The IBD diagnosis was based on the response to the mailed-out questionnaire (328 IBD mothers), with the addition of 174 mothers who were recorded as having IBD by the Norwegian Patient Registry (NPR). After excluding multiple births and FFQ’s with invalid energy reports and −30 kg > GWG > 50 kg, 342 IBD mothers, 148 with CD and 194 with UC, were eligible for the analyses of the impact of PPDS on the association between IBD and GWG or SGA (Fig. 1, flow chart). Using the same exclusion criteria for non-IBD, *n* = 68,858 mothers were available as controls.

### Dietary information

Dietary information in this study was obtained by a new semi-quantitative questionnaire FFQ used in MoBa from March 2002 and onwards. From 1999 to 2002, participants answered a general FFQ that had not been validated for pregnant women and calculations based on this FFQ could therefore not be included in our analyses. This MoBa FFQ was developed and validated especially for pregnant women in the MoBa study and has been validated in a sub-sample of the cohort participants using 4 days weighed food record and biological markers of intake as reference measures [19]. Intake of dairy products by the MoBa FFQ has been validated using 24-h urinary iodine excretion as a biomarker [20]. We used FoodCalc [21] and the Norwegian food composition table to calculate daily intakes of food, nutrients and energy assuming standard portion sizes. Food items included in the analyses of protein from dairy sources are listed in Table 1.The participants were asked to report any changes of diet related to pregnancy. As for dairy products, they were asked if the current intake was lower, higher or as before pregnancy. Misreporting was handled by consistency checks, and reports with invalid daily energy intake were excluded (4.5 < MJ or MJ < 20) [22]. The quantity and the proportion of protein from dairy sources were calculated, and the calculated PPDS was ranked in quartiles. The two lowest quartiles were merged and considered to represent the lowest of three PPDS groups. The ranges of PPDS in each group from lowest to highest are: 0.0–0.21, 0.21–0.27, 0.27–0.93.

**Table 1:** The intake of protein from dairy products in IBD and non-IBD mothers, from MoBa 2002–2008

	Non-IBD	IBD	CD	UC
Mean gram protein (SD)				
Butter	0.033 (0.08)	0.033 (0.08)	0.033 (0.08)	0.034 (0.08)
White cheese high caloric	3.64 (3.8)	3.8 (4.2)	3.2 (3.7)	4.2 (4.6)
White cheese low caloric	0.46 (1.5)	0.39 (1.59)	0.42 (1.97)	0.36 (1.2)
Blue cheese	0.04 (0.02)	0.027 (0.16)	0.026 (0.12)	0.028 (0.19)
Gudbrand Valley cheese	0.68 (1.1)	0.62 (1.05)	0.5 (0.86)	0.70 (1.18)
Milk products - high caloric	1.2 (3.7)	1.1 (2.8)	0.99 (2.6)	1.1 (2.9)
Milk products - low caloric	9.9 (10.3)	6.33 (8.3)	6.48 (7.8)	6.2 (8.7)
Biola	1.1 (3.0)	1.1 (2.8)	1.2 (3.3)	0.97 (2.4)
Yoghurt	2.3 (3.4)	2.1 (4.0)	2.2 (4.9)	2.0 (3.1)
Total dairy products	19.4 (12.7)	15.4 (11.9)	15.0 (12.2)	15.6 (11.6)

Plural birth and extreme energy intake and 50 kg < GWG < − 30 kg excluded

### Outcomes variable

SGA was defined as birth weight below the 10th percentile of population-based birthweight, based on gender and week of gestation. Gestational age was based on the first trimester estimation by ultrasound, or on the last menstrual period, if the measure from the ultrasound examination was missing (1.7%).

### Other variables

GWG was based on self-reported prepregnant weight recorded in the baseline questionnaire (Q1) and self-reported weight at delivery recorded in the fourth questionnaire 6 months after birth (Q4). The classification of inadequate GWG is dependent on the prepregnant body mass index (BMI) and based on recommendations by The US Institute of Medicine (IOM), which has been adopted by The Norwegian Directorate of Health (Table 2) [23].

**Table 2:** The American Institute of Medicine (IOM) recommendations for gestational weight gain (GWG)

Prepregnant categories of BMI (kg/m^2^)	GWG (kg) according to IOM recommendations
BMI < 18.5	12.5 < GWG < 18
18.5 < BMI < 24.9	11.5 < GWG < 15
25 < BMI < 29.9	7 < GWG < 11.5
BMI > 30	5 < GWG < 9

Information about smoking status was obtained from Q1 at week 15–17, shortly before the first ultrasound examination, defined in terms of non-smokers, occasional smokers and daily smokers, the latter two merged and considered to represent the smokers.

Education level was divided in three categories by length of education; ≤ 12 years, 13–16 years, ≥ 17 years. Maternal diabetic condition, recorded as a dichotomous variable, included diabetes I and II as well as gestational diabetes. Maternal hypertension was defined as; systolic blood pressure ≥ 140 mmHg, or diastolic blood pressure ≥ 90 mmHg (Table 3).

**Table 3:** Descriptive data, mothers’ disease, smoking history and pregnancy outcomes in maternal IBD compared to controls, from MoBa 2002–2008

		Non-IBD (%)	IBD (%)	*P-value	CD (%)	*P-value	UC (%)	*P-value
PPDS, ranked in quartiles (q1-q4)	Group 1 (q1 + q2) (0.0–0.21)	34,241(49.8)	222 (64.9)	< 0.001	95 (64.2)	< 0.001	127 (65.5)	< 0.001
	Group 2 (q3) (0,21-0,27)	17,516 (25.5)	70 (20.5)		35 (23.6)		35 (18.0)	
	Group 3 (q4) (0,27–0.93)	17,068 (24.8)	50 (14.6)		18 (12.2)		32 (16.5)	
Age	N (mean, std)	30.3 (4.5)	30.6 (4.2)	0.16	30.1 (4.5)	0.62	31.0 (4.1)	0.02
Education	High school or less	21,254 (32.4)	98 (30.5)	0.12	47 (33.8)	0.32	51 (28.0)	0.18
	College 3 years	27,966 (42.7)	155 (48.3)		65 (46.8)		90 (49.5)	
	Master or higher	16,278 (24.9)	68 (21.2)		27 (19.4)		41 (22.5)	
Diabetes	yes	981 (1.4)	3 (0.9)	0.34	1 (0.5)	0.44	2 (1.0)	0.65
	no	67,844 (98.6)	339 (99.1)		182 (99.5)		192 (99.0)	
Hypertension	yes	3958 (5.8)	18 (5.3)	0.90	10 (5.5)	0.86	10 (5.2)	0.72
	no	64,867 (94.2)	324 (94.7)		173 (94.5)		184 (94.8)	
Smoking history	Current	4680 (7.5)	25 (7.9)	0.59	22 (12.7)	0.032	8 (4.5)	0.14
	Never	58,083 (92.5)	291 (92.1)		151 (87.3)		169 (95.5)	
BMI categories	BMI < 18.5	1963 (2.9)	12 (3.5)	0.28	7 (3.9)	0.24	6 (3.1)	0.78
	18.5 ≤ BMI < 24.9	45,202 (65.8)	238(69.8)		123 (69.1)		133 (68.9)	
	25 ≤ BMI < 29.9	15,086 (22.0)	68 (19.9)		37 (20.8)		39 (20.2)	
	BMI > 30	6429 (9.4)	23 (6.7)		11 (6.2)		15 (7.8)	
GWG	N (mean, std)	68,680 (14.8, 9.2)	342 (13.8, 6.3)	0.049	13.5 (6.8)	0.09	14.07 (5.9)	0.26
Inadequate GWG	yes	13,211 (19.2)	104 (30.5)	< 0.001	52 (35.1)	< 0.001	52 (26.9)	0.007
	no	55,469 (81.8)	237 (69.5)		96 (64.9)		141 (73.1)	
SGA	yes	4425 (6.5)	31 (9.1)	0.03	15 (10.1)	0.07	16 (8.2)	0.31
	no	64,091 (93.5)	311 (90.8)		133 (89.9)		178 (91.8)	
Disease activity	yes	N/A	58 (13.7)		16 (8.7))		42 (17.5)	
	no	N/A	270 (86.3)		120 (91.3)		150 (82.5)	
Total energy intake - kcal	N (mean, std)	2298 (601)	2285 (607)	0.70	2319 (663)	0.66	2258 (587)	0.37
BMI continuous	N (mean, std)	24.0 (4.2)	23.5 (3.8)	0.02	23.2 (3.9)	0.026	23.7 (3.8)	0.29

*BMI* Body mass index, *GWG* Gestational weight gain, *PPDS* Proportion of protein from dairy sources, *SGA* Small for gestational age

Plural birth, extreme energy intake and 50 kg < GWG < −30 kg excluded *p-values of chi squared tests comparing categorical variables between non-IBD and IBD/CD/UC

### Statistical analyses

Kruskal-Wallis tests were used to compare continuous variables between the groups and Chi-squared tests for categorical variables.

We used logistic regression analyses to model multivariate associations. First, models were fitted to estimate the relationship between PPDS and inadequate GWG in IBD compared to non-IBD mothers. Next, to study the effect of PPDS on the association between SGA and IBD, four different logistic regression models were fitted by adding variables in the following way: Model 1: PPDS groups, with highest intake as reference group; Model 2: the interaction term IBD-control by PPDS groups (IBD/PPDS), with high PPDS group as the reference group; Model 3: inadequate GWG; and Model 4: an interaction term between IBD and inadequate GWG. In all models, we adjusted for maternal age, education level, smoking status, chronic diseases (hypertension and diabetes mellitus), energy intake and BMI.

We depict the non-linear association between SGA and PPDS or GWG using cubic splines. These graphs show the log odds for SGA as a function of PPDS and GWG adjusting for mothers’ age, education, current smoking. We show risk profiles for IBD mothers and non-IBD mothers.

We report adjusted odds ratios (OR) and corresponding 95% confidence intervals (CIs). *P*-values less than 0.05 were considered statistically significant. The statistical analyses were performed using the software SPSS version 23 and R version 3.5.

## Results

Three hundred and forty-two IBD mothers, 148 CD and 194 UC mothers, and 68,858 non-IBD mothers were available for the analyses exploring the impact of PDDS on the association between IBD and SGA. The analyses of inadequate GWG included one less, 341 IBD mothers, because one UC mother lacked information about BMI (Table 4).

**Table 4:** Inadequate GWG by PPDS groups and ORs for inadequate GWG in IBD compared to non-IBD, from MoBa 2002–2008

		PPDS groups^a^			ORs for inadequate GWG in IBD compared to non-IBD with low intake of PPDS	
	N (obs)	1	2	3	aOR (95% CI) low compared to high PDDS	aOR (95%) middle compared to high PPDS
Inadequate GWG (%)						
Non-IBD	68,680	6923 (20.3)	3221 (18.4)	3067 (18)		
IBD	341	68 (30.8)	22 (31.4)	14 (28)	2.35 (1.23, 4.49)	1.38 (0.64, 2.99)
CD	148	31 (32.6)	17 (48.6)	4 (18)	4.22 (1.28, 13.92)	4.09 (1.13, 14.29)
UC	193	37 (29.4)	5 (14.3)	10 (31.1)	1.65 (0.76, 3.59)	0.45 (0.14, 1.51)

OR adjusted for mothers’ education and age, ^a^Group 1 = quartile 1 + 2, group 2 = quartile 3, group 3 = quartile 4, highest PPDS group (group 3) was used as reference group, *GWG* gestational weight gain, *PPDS* proportion of protein from dairy sources

Plural birth and extreme energy intake and 50 kg < GWG < −30 kg excluded

### The proportion of protein from dairy sources (PPDS) in IBD and non-IBD mothers

Of the IBD mothers who answered the FFQ, 56.4% reported that their current intake of dairy products was as before and 32.5% higher than before pregnancy.

**Fig. 2:**
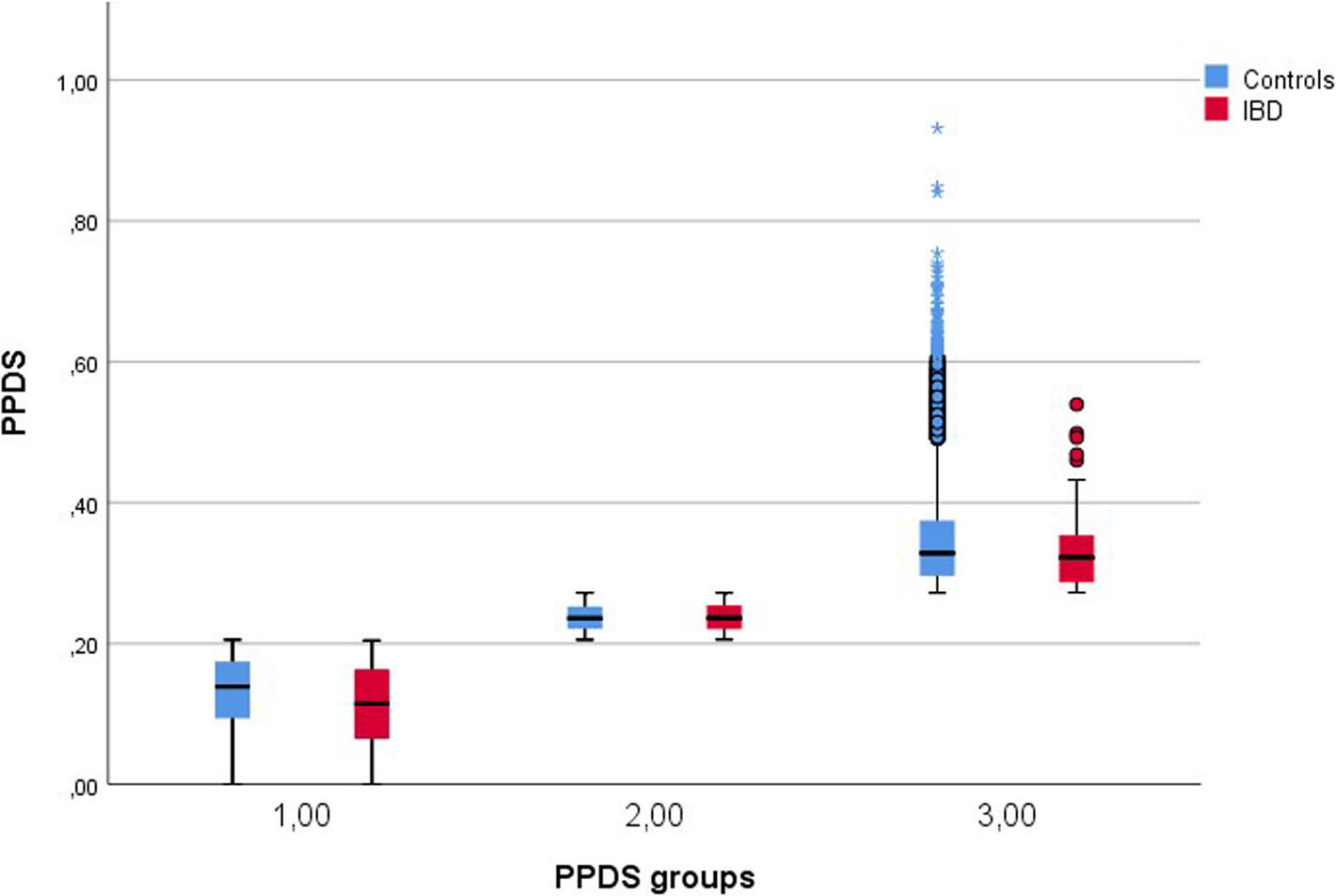
Difference in proportion of protein from dairy sources (PPDS) between IBD and non-IBD, by PPDS-groups. Legend: The boxplots compare the distribution of PPDS between IBD and non-IBD mothers in each PPDS groups. The difference in PPDS between IBD and non-IBD mothers is significant only in the lowest PPDS group

The total mean gram protein from dairy sources was 19.38 in non-IBD mothers and 15.38 in IBD mothers (*p* < 0.001) (Table 1). The mean values of PPDS were 0.17 (range 0–0.50) and 0.21 (range 0–0.93) in IBD and non-IBD mothers, respectively (*p* < 0.001). The odds of belonging to the lowest compared to the highest PPDS group were doubled in IBD mothers adjusted for maternal age and education level [aOR = 2.02 (95% CI: 1.53, 2.67)]. The corresponding odds in CD and UC mothers were aOR = 2.29 (95% CI:1.48, 3.57 and aOR = 1.79 (95% CI:1.26, 2.56), respectively. The difference in PPDS between IBD and non-IBD mothers was significant only in the lowest PPDS group (group 1: *p* < 0.001, group 2: *p* = 0.890, group 3: *p* = 0.100) (Fig. 2). A similar pattern was found comparing CD or UC mothers with non-IBD mothers (not shown).

### PPDS and risk of inadequate GWG in IBD compared to non- IBD mothers

IBD mothers with low compared to high PPDS had twice as high risk of inadequate GWG than non-IBD mothers (aOR = 2.35 (95% CI: 1.23, 4.49). CD mothers were the ones who contributed to this significant higher risk of inadequate GWG in IBD. CD mothers with low and middle PPDS, using high PPDS as the reference group, had a fourfold risk of inadequate GWG compared to non-IBD mothers [aOR = 4.22 (95% CI: 1.28, 13.92) and OR = 4.09 (95% CI: 1.13, 14.29, respectively)] (Table 4).

### PPDS and risk of SGA in IBD mothers compared to non-IBD mother

Four regression models with SGA as the outcome variable and IBD as the predictor, explored the impact of PPDS on the association between SGA and IBD compared to non-IBD (Table 5). PPDS with the highest PPDS group as the reference-group was added to Model 1 in addition to possible confounders such as mothers’ age, education, current smoking and chronic diseases (diabetes and hypertension).

**Table 5:** SGA by PPDS groups and ORs for SGA in IBD mothers compared to non-IBD, from MoBa 2002–2008

		Distribution of SGA in PPDS groups^a^			Odds ratios of SGA in IBD mothers compared to non-IBD			
	N (obs)	1	2	3	OR (95% CI)^b^ Model 1	aOR (95% CI)^c^ Model 2	aOR (95% CI)^d^ Model 3	aOR (95% CI)^e^ Model 4
Non-IBD	68,858	2261 (6.8)	1129 (6.5)	1035 (6.3)				
IBD	342	12 (6.9)	7 (8.2)	12 (19.7)	1.48 (0.99, 2.19)	4.50 (2.17, 9.34)	4.26 (2.04, 8.89)	3.17 (1.38, 7.29)
CD	148	8 (6.3)	5 (14.3)	5 (22.2)	1.29 (0.97, 1.70)	2.08 (1.16, 3.73)	2.10 (1.17, 3.77)	1.92 (1.03, 3.58)
UC	194	11 (4.7)	2 (5.7)	8 (25)	1.34 (0.77, 2.32)	4.59 (1.82, 11.63)	4.14 (1.61, 10.64)	2.91 (0.93, 9.09)

^a^PPDS groups derived by ranking PPDS into quartiles (q1-q4) with group 1 = q1 + q2, group 2 = q3, group 3 = q4. ^b^Model adjusted for education, mothers age and chronic disease (diabetes mellitus and hypertension), smoking status, ^c^interaction term IBD/PPDS included, highest PPDS group as reference group, ^d^inadequate GWG included, ^e^interaction term IBD/GWG included. *GWG* Gestational weight gain, *PPDS* Proportion of protein from dairy sources, *SGA* Small for gestational age

Plural birth, extreme energy intake and extreme GWG (50 kg < GWG < -30 kg) excluded

The interaction term IBD/PPDS was added to Model 2 because of its significant association with SGA. The interaction term revealed that IBD mothers with low PPDS had a significantly lower risk of SGA than non-IBD mothers and IBD mothers with high PPDS (aOR = 0.19, 95% CI: 0.07, 0.50). By adding this interaction term, IBD/PPDS, to regression Model 2, the odds for SGA in IBD compared to non-IBD increased with a factor of three, from OR = 1.48 (95% CI: 0.99, 2.19) to OR = 4.50 (95% CI: 2.17, 9.34) (Table 5). Inadequate GWG was added in Model 3 as a possible confounder, because of the strong association between inadequate GWG and SGA and between inadequate GWG and IBD (data not shown) [15]. Furthermore, since the interaction term IBD/inadequate GWG associated with SGA as a trend (*p* = 0.069), it was implemented in Model 4, shown in the last column in Table 5.

**Fig. 3:**
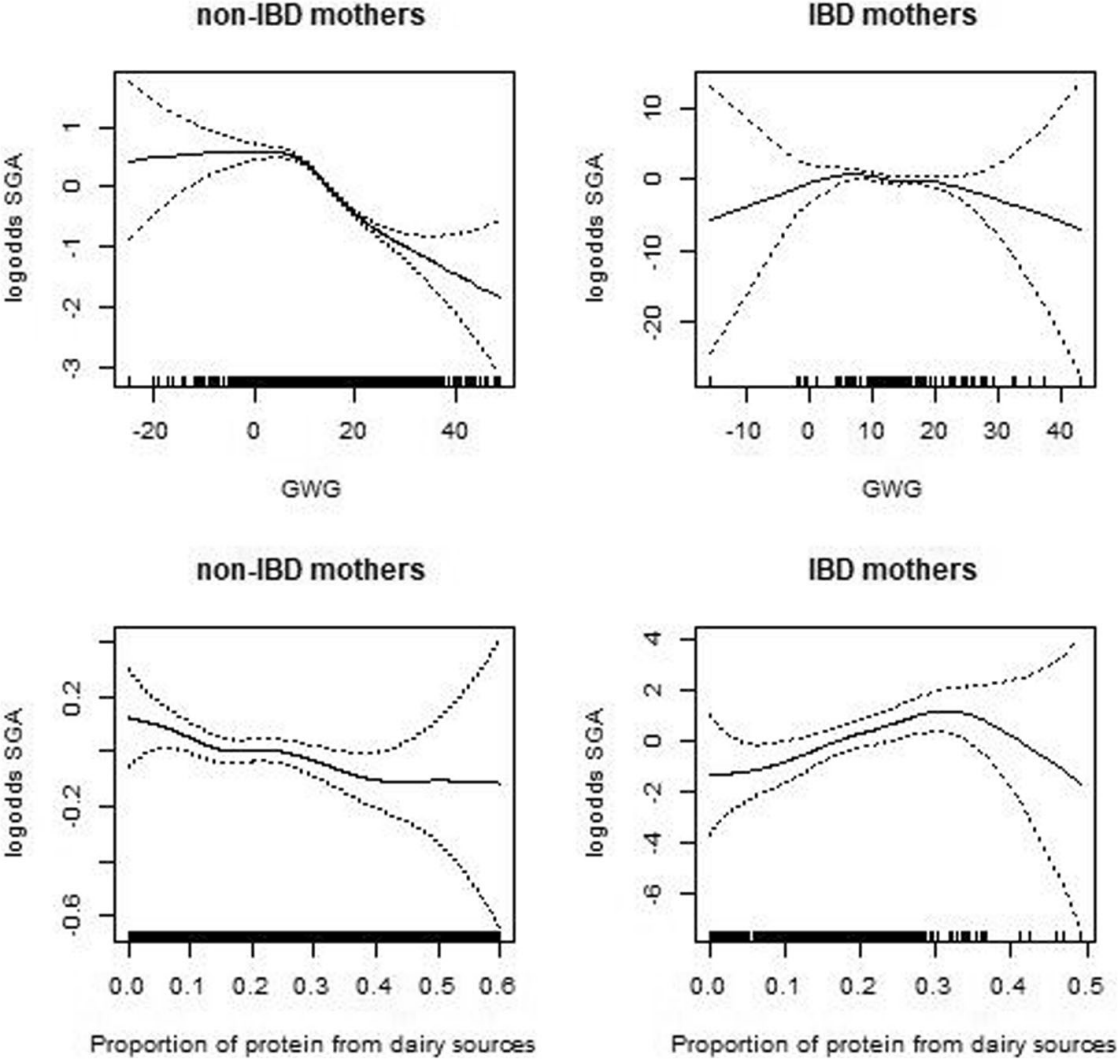
Risk functions of SGA dependent on GWG or PPDS in IBD and non-IBD mothers. Legend: Risk functions of SGA with 95% CI bounds in non-IBD (left) and IBD mothers (right). The top figures show the risk functions of SGA dependent of GWG and the lower figures the risk functions of SGA dependent of PPDS, adjusted for mothers’ age, education, and current smoking. GWG is negatively associated with risk of SGA in both IBD and non-IBD mothers. The risk of SGA is negatively associated with PPDS in non-IBD mother. In contrst, the risk of SGA is positively associated with PPDS in IBD mothers within the range of PPDS (0–0.35), which includes the majority of IBD mothers. The huge confident interval linked to PPDS >0.35 reflects the uncertainty of the risk profile of SGA in IBD mothers with a high level of PPDS due to the small number of IBD mothers

The risk of SGA in IBD compared to non-IBD sustained significant in model 3 and 4. A similar pattern of SGA risk was found in CD mothers; the risk of SGA remained unchanged in Model 4 (OR = 1.92, 95% CI:1.03, 3.58). This was not the case for UC-mothers. The association between risk of SGA and UC mothers changed from non-significant in Model 1 to significant in Model 2 but did not sustain in Model 4 (*p* = 0.066) after adjusting for inadequate GWG and the interaction term IBD/inadequate GWG. Figure 3 shows the risk profile of SGA as a function of GWG or PPDS in IBD and non-IBD mothers. The range of PPDS was wider for non-IBD than for IBD mothers. The PPDS range of the IBD mothers from 0 to 0.35, which included the majority of the IBD mothers, shows a positive association with the risk of SGA. In contrast, PPDS in non-IBD mothers shows a negative association with the risk of SGA. GWG was negatively associated with SGA in both IBD and non-IBD mothers.

## Discussion

The present study hypothesized that IBD mothers had lower PPDS than non-IBD mothers, and furthermore, that the assumed restriction of PPDS in IBD would increase their risk for both inadequate GWG and SGA. We did find significantly lower PPDS in IBD than in non-IBD mothers, and further, that low compared to high PPDS increased the risk of inadequate GWG in IBD mothers. Surprisingly, in opposite to our hypothesis, low PPDS in IBD mothers was associated with a lower likelihood of SGA than in non-IBD and IBD mothers with high PPDS.

The risk of belonging to the lowest PPDS group was twice as high in IBD mothers than in non-IBD mothers (aOR = 2.02, 95% CI: 1.53, 2.67). This finding indicates that avoidance of dairy products is as common in pregnancies with IBD as in IBD patients in general [17, 24, 25], and that factors associated with adherence to diet are similar before and during pregnancy [26]. An investigation from Iceland [17] showed that as much as 87% of the IBD patients claimed that diet affected gastrointestinal symptoms, and accordingly, 60% restricted the intake from dairy sources. Disease activity is an essential factor influencing restriction of dairy intake, probably caused by a transitory lactose intolerance [10]. However, investigations have demonstrated that a substantial part of IBD patients avoid dairy products also in remission due to abdominal symptoms, food belief and dietary advice [25, 27–29].

Milk and dairy intake during pregnancy is a subject of great interest because of its nutritive value for both maternal weight gain during pregnancy and for fetal growth [30–32].

Like results from two European birth cohorts [33, 34], we found a negative association between dairy intake and the risk of SGA in the general population (Fig. 3). Surprisingly, and in opposition to the non-IBD mothers, the risk of SGA in IBD mothers showed a positive association; low PPDS was associated with low risk of SGA. These results mirror the results from the regression analyses exploring the impact of PPDS on the association between SGA and IBD, compared to non-IBD (Table 5). The interaction term IBD/PPDS revealed that IBD mothers with low PPDS had a reduced risk of SGA compared to non-IBD and IBD mothers with high PPDS (OR = 0.19, 95% CI: 0.07, 0.50). Further, this interaction term IBD/PDDS appeared to be the contributing factor that significantly linked SGA to IBD (Models 2, 3, 4, Table 5).

We hypothesized the opposite scenario, that low PPDS, possible by its link to inadequate GWG, was one of the contributing factors that increased the risk of SGA in IBD compared to non-IBD. However, based on our results, the positive association between risk of SGA and intake of dairy products in IBD, in contrast to non-IBD, we speculate that unrecognized lactose malabsorption frequently coexists with IBD. Lactose malabsorption leads to pass-over of unabsorbed sugars to the colon where microflora fermentation results in gases, such as hydrogen and methane, and a variety of bacterial metabolites. These products of the anaerobe metabolism have been proposed to cause cramping diarrhea by inducing Ca^2+^ signalling mechanisms in the gut bacteria, influencing bacterial growth, analog to the diarrhea in severe gut infection [35]. The delay in onset (24 h) and duration of diarrhea (48 h) after lactose challenge demonstrated in several studies, could not be explained by the osmotic effect of lactose alone [36, 37]. We believe that diarrhea induced by high PPDS intake in IBD mothers with lactose malabsorption has a negative impact on maternal nutrition and fetal growth in pregnant women with IBD who are already prone to malnutrition and weight loss [12]. Moreover, a possible coexisting lactose malabsorption has persisted for an extended period, with further negative consequences for maternal nutritional status, since approximately 90% of the IBD mothers in our cohort reported that the intake of dairy products during pregnancy was as high as, or higher than before pregnancy.

Several studies have demonstrated that lactose intolerance is not more frequent in IBD patients in remission than in healthy controls using a breath hydrogen test [38, 39]. However, using only breath hydrogen test alone detects < 50% of those who are lactose sensitive [36, 40]. One study demonstrated lactose sensitivity in 68% of UC patients, 76% of CD patients, all in remission, and none in healthy controls, using comprehensive tests of lactose sensitivity [37]. The authors believed that the reason for the high prevalence of lactose sensitivity in IBD patients in that study was the use of comprehensive lactose sensitivity tests. The tests included the patient’s genotype, breath hydrogen (>20 ppm over the nadir) and methane (>5 ppm over the nadir) measurements, and/or the occurrence of the gut and systemic symptoms, after a lactose challenge of 50 g.

Lactose malabsorption refers to any cause of failure to absorb or digest lactose. Lactase deficiency secondary to small intestine diseases, such as bowel resections, inflammation, short gut transit time and intestinal bowel overgrowth (SIBO), are all relevant for IBD, and especially for CD [41, 42]. Further, the likelihood of developing symptoms related to consumption of dairy products also depends on the presence of IBS, which often coexist with IBD [43].

In a recently published MoBa-IBD study, we showed that IBD mothers had a higher risk of inadequate GWG than non-IBD mothers, which doubled their risk of SGA compared to non-IBD mothers [15]. The present study emphasizes the importance of PPDS intake for inadequate GWG in CD. Low and middle PPDS compared to high PPDS was a fourfold stronger predictor for inadequate GWG in CD, compared to non-IBD (Table 4). Low PPDS was associated with both low risk of SGA and inadequate GWG in CD, which partly explains why inadequate GWG did not confound the association between risk of SGA and CD compared to non-IBD (Model 3 and 4 in Table 5). This was not the case for UC. The distribution of inadequate GWG in the PPDS groups differed between CD and UC. The proportion of inadequate GWG in the highest PPDS group was 31% in UC compared to 18% in CD, suggesting that other factors than low PPDS contribute to inadequate GWG, especially in UC. Low PPDS was not associated with inadequate GWG in UC (Table 4), and probably the reason why inadequate GWG represents a confounder to the association between risk of SGA and UC compared to non-IBD (*p* = 0.066) (Model 4, Table 5).

### Strengths and limitations

The strengths of this study include the large and nationwide sample size and the linkage to the medical birth registry [28]. Based on the questionnaires and MBRN we were able to include relevant potential confounders. Three hundred and forty-one IBD mothers were available for the SGA analyses, and CD and UC were analyzed separately. Although the number of included IBD mothers is low, the number of IBD mothers is accord with the background population [44].

This study has major limitations in its methodology like recall bias in obtaining dietary information, lack of information regarding disease activity and use of IBD drugs, all factors known to influence the GWG or SGA. The use of FFQ, which covers the first four to 5 months of pregnancy, introduces both recall bias and averaging. Although adherence to dietary pattern has been demonstrated to stable during pregnancy [30], we might not have obtained the actual intake of PPDS in the last part of the pregnancy. The prospective design of the study with dietary assessment in mid-pregnancy to obtain the exposure prior to the pregnancy outcome [6] minimizes the potential misclassification of diet. Furthermore, because the information of SGA was obtained from the MBR, not connected to the FFQ, the differential misclassification of SGA related to the intake of dairy products is unlikely. Unfortunately, we were not able to obtain reliable information about disease activity before and during pregnancy in the present study. The IBD-mothers was not followed prospectively through their pregnancy with clinical examination and biomarkers such as CRP and calprotectin. Disease activity has been shown to significantly reduce the intake of dairy products [27] but is also a predictor of inadequate GWG [45]. Based on the results from these studies, we can only speculate that disease activity might have attenuated the association between IBD and SGA by its link to both low PPDS and inadequate GWG.

## Conclusions

This study revealed that PPDS was lower in IBD mothers than in non-IBD mothers and, for the first time, to the best of our knowledge, that low PPDS in IBD is associated with reduced risk of SGA compared to non-IBD and IBD mothers with high PPDS.

Furthermore, this reduced risk of SGA in IBD with low compared to high PPDS was the factor that linked SGA to IBD compared to non-IBD.

Our findings indicating a link between high intake of dairy products and increased risk of SGA in IBD mothers, and possibly underlying factors such as lactose malabsorption, has to be examined in future investigations.

## Data Availability

The consent given by the participants does not open for storage of data on an individual level in repositories or journals. Researchers who want access to data sets for replication should submit an application to datatilgang@fhi.no. Access to data sets requires approval from the Regional committees for medical and health research ethics in Norway and a formal contract with MoBa.

## Abbreviations

BMI: Body mass index
CD: Crohn’s disease
CI: Confidence interval
FFQ: Food frequency questionnaire
GWG: Gestational weight gain
IBD: Inflammatory bowel disease
IOM: Institute of Medicine
MBRN: Medical birth registry of Norway
MoBa: The Norwegian Mother, Father and Child cohort Study
NPR: The Norwegian Patient Registry
OR: Odds ratio
PPDS: The proportion of protein from dairy sources
SGA: Small for gestational age
UC: Ulcerative colitis
